# Developing a Curriculum for Information and Communications Technology Use in Global Health Research and Training: A Qualitative Study Among Chinese Health Sciences Graduate Students

**DOI:** 10.2196/mededu.6590

**Published:** 2017-06-12

**Authors:** Zhenyu Ma, Li Yang, Lan Yang, Kaiyong Huang, Hongping Yu, Huimin He, Jiaji Wang, Le Cai, Jie Wang, Hua Fu, Lisa Quintiliani, Robert H Friedman, Jian Xiao, Abu S Abdullah

**Affiliations:** ^1^ School of Public Health Guangxi Medical University Nanning China; ^2^ School of Information and Management Guangxi Medical University Nanning China; ^3^ School of Public Health Guangzhou Medical University Guangzhou China; ^4^ School of Public Health Kunming Medical University Kunming China; ^5^ School of Public Health Fudan University Shanghai China; ^6^ Boston University School of Medicine Department of Medicine Boston Medical Center Boston, MA United States; ^7^ School of Medicine Guangxi Univeristy of Chinese Medicine Nanning China; ^8^ Duke Global Health Institute Duke University Durham, NC United States; ^9^ Duke Kunshan University Global Health Program Kunshan China

**Keywords:** information and communication technology, ICT, global health, research, training, China

## Abstract

**Background:**

Rapid development of information and communications technology (ICT) during the last decade has transformed biomedical and population-based research and has become an essential part of many types of research and educational programs. However, access to these ICT resources and the capacity to use them in global health research are often lacking in low- and middle-income country (LMIC) institutions.

**Objective:**

The aim of our study was to assess the practical issues (ie, perceptions and learning needs) of ICT use among health sciences graduate students at 6 major medical universities of southern China.

**Methods:**

Ten focus group discussions (FGDs) were conducted from December 2015 to March 2016, involving 74 health sciences graduate students studying at 6 major medical universities in southern China. The sampling method was opportunistic, accounting for the graduate program enrolled and the academic year. All FGDs were audio recorded and thematic content analysis was performed.

**Results:**

Researchers had different views and arguments about the use of ICT which are summarized under six themes: (1) ICT use in routine research, (2) ICT-related training experiences, (3) understanding about the pros and cons of Web-based training, (4) attitudes toward the design of ICT training curriculum, (5) potential challenges to promoting ICT courses, and (6) related marketing strategies for ICT training curriculum. Many graduate students used ICT on a daily basis in their research to stay up-to-date on current development in their area of research or study or practice. The participants were very willing to participate in ICT courses that were relevant to their academic majors and would count credits. Suggestion for an ICT curriculum included (1) both organized training course or short lecture series, depending on the background and specialty of the students, (2) a mixture of lecture and Web-based activities, and (3) inclusion of topics that are career focused.

**Conclusions:**

The findings of this study suggest that a need exists for a specialized curriculum related to ICT use in health research for health sciences graduate students in China. The results have important implications for the design and implementation of ICT-related educational program in China or other developing countries.

## Introduction

### Background

Information and communications technology (ICT) can be defined as tools that facilitate communication and the processing and transformation of data by electronic means [1]. The application of ICT tools in research and training in the field of health sciences, including biomedical and population-based programs, has grown dramatically in recent years. ICT has transformed the way health care is delivered and health-related research is conducted. Meanwhile, more training programs to use ICT are delivered in developed and developing communities. The benefits of ICT use include [2]: (1) improve dissemination of public health information and facilitate public discourse around subjects that are major public health threats, (2) enable collaboration and cooperation among health workers, (3) support more effective health research and the dissemination and access to research findings, (4) improve the efficiency of health administration, and (5) improve the ability to monitor outbreaks and have effective management plans. During the last two decades, the use of ICT has contributed significantly in conducting biomedical and population-based research, improving health sector service provision and promoting health in many developed countries [3-8].

### Information and Communications Technology

Within the health care context, information flow between health care providers and from health care providers to health consumers is crucial [9]. At the same time, global health research projects that are distributed across multiple countries encourage collaborations and networking for data sharing and new forms of research training. The use of ICT is crucial in expediting such global health initiatives [10], especially to address needs in the areas of health care and research, and training in low- and middle-income countries (LMICs) [11]. Building institutional capacity to create internal resources to accelerate ICT use in training and research programs is an important first step toward building global health research and training programs. However, in a developing country setting, the critical mass of professional and community users of ICTs in health generally has not yet been reached in many sectors, let alone the health sector [12]. It is in the developing world that ICTs can and should make the highest impact. This is because these populations probably require most of the benefits that are provided by the use of ICTs, such as ready access to needed clinical expertise to facilitate better diagnostic and therapeutic decisions, and ICT-based research expertise to carry out population-based or clinical research. The purpose of this study was to identify practical issues (ie, perceptions, learning needs) in developing an ICT curriculum to be used by graduate level researchers at major universities in southern China. We gathered information from graduate students, including masters and doctoral level research focused students, because they are the population who are currently engaged (or in the future will engage) in research and are in a good position to describe their perspective.

## Methods

### Sample and Settings

Participants were graduate students of health sciences programs (ie, population health or biomedicine) from the selected 6 major medical universities in southern China covering 4 provinces. These universities were Guangxi Medical University (GXMU), Guangxi University of Chinese Medicine (GXTCMU), Guilin Medical University (GLMU), Guangzhou Medical University (GZHMU), Kunming Medical University KMMU), and Fudan University (FDU).

### Methods of Subject Recruitment

Participants were conveniently recruited during December 2015 to March 2016 in each of the participating universities. A liaison person who served as a senior researcher in each of the universities recruited subjects for focus group discussions. Selection criteria were as follows: (1) current graduate (masters or doctoral) student of any of the health sciences programs at the University who responded to our subject recruitment announcement within 1 week time frame and (2) willing to give consent to participate in the focus groups. Once we had reached our target sample size for the focus groups, we stopped recruiting.

### Data Collection

A semi-structured FGD guide was developed with reference to the research team’s earlier work [13,14] and pilot tested with 7 graduate students resulting in minor changes. All of the FGDs were conducted in Mandarin Chinese and audio recorded. The guide included questions on the following areas: major area of research, a definition of ICT, types of researchers who should study the application of ICT, whether ICT training is useful, past ICT-related training experiences, and pros and cons of Web-based courses. Interviewers were graduate students at the School of Public Health of Guangxi Medical University, and attended a 2-day training course designed for them. The training described the overall research projects, including logistical issues, recruitment process, familiarization with the FGD guide, and description of ICT and ICT-related research in areas of health sciences programs. The training also included a session on the ethical aspects of human subject research. Two interviewers worked as a team to collect data; one moderated the focus group and the other took detailed notes and also recorded the session with a digital voice recorder (after permission had been obtained from the participants). All focus groups were held in a private meeting room within the university and lasted for approximately 90 min. The sessions started with the moderator explaining the purpose of the group discussion and assuring confidentiality of the data collected for the research project. To compensate participants for their time, each participant was given a cash amount of RMB 50 (US $8). Written informed consent was obtained from each participant. The study was approved by the Ethics Committee of the Guangxi Medical University (No. IRB-SPH-2015: 009).

### Analyses

The interviewers discussed and summarized the main content of each focus group and reviewed the notes taken immediately after the focus group. These debriefings were useful (1) to identify the most crucial themes and ideas and (2) to evaluate the demand for possible modification in the subsequent focus group. The audio recordings were reviewed and transcribed for each group during the translation of Chinese to English. Two members of the research team coded each transcript independently, with discrepancies resolved through consensus. The process of coding involved identifying central themes and highlighting these on the transcripts [15]. All additional notes taken during the course of focus group were examined to identify diversified themes presented in theses qualitative discussions. No specific software was used for the data analysis due to the small volume of the data. Rather, line-by-line coding, categorization, and theme extraction were used to conduct the content analysis [16].

## Results

### Participant Characteristics

A total of 10 FGDs were conducted involving 74 graduate students (Table 1).

Of the participants, 64% (48/74) were females and 53% (39/74) were first-year graduate students. Participants were affiliated with across the disciplines of population health or public health (42%, 31/74), clinical medicine (34%, 25/74), basic science (11%, 8/74), and others (13%, 10/74). The research background and areas of studies of the students varied across the university (Table 1).

The findings revealed the following 6 themes relating to the use of ICT: (1) ICT application in routine research, (2) ICT-related training experience, (3) understanding about pros and cons of Web-based training, (4) attitude toward the design of ICT training courses, (5) potential challenges to ICT course promotions, and (6) related marketing strategies for ICT training curriculum. These themes are described in the following section and supplemented by participants’ statements on key themes provided in Table 2.

### Information and Communications Technology (ICT) Application in Routine Research

Regarding ICT application in daily research, almost all (72/74) participants described the irreplaceable role of ICT to facilitate their academic work. They discussed using ICT for information seeking to data gathering to maintaining communication. For example, one of the participants mentioned:

It truly brings great convenience to scientific research, we track and get information about the latest findings in our field of research and pursue progress by searching scientific literature in the PubMed.

Another graduate student said:

We are so addicted in it and cannot survive without technology. Computer andInternetuse have become part of student’s daily life.For example, we use WeChat to share and seek information. We use texting,email with professional and even surfInternetand download papers with ICT.Ihave no idea where my experiment would go without the help of some specific technology.

Use of ICT tools to check emails was common among the participants with about half (37/74) checking their emails daily and few (9/74) checking at least weekly.

Graduate students thought that ICT brings convenience to their research by synthesizing information and ensuring accuracy. One of the interviewees said:

Most of the ICT-related skills are easy to acquire and not that professional, I wonder whether there are some authoritative ICT skills can benefit our research.

One of the students added:

The desire to acquire skills could drive us to take the initiatives of self-guided learning.

### ICT-Related Training Experiences

Less than half (43%, 32/74) of the participants took some training course relating to ICT. The following courses were mentioned very often: basic computer programming and R language, PPT-training (ie, how to make presentation in a scientific meeting or in the group), PubMed for searching scientific literature, Medical Statistics (a required course for most medical universities in China, Web-based course training such as CET-4 or CET-6 (an English test for Chinese college students), and the Party lecture (delivering basic theories of the Communist Party of China by lectures). Participants’ willingness to attend professional ICT course varied across the universities. For example, all students of GLMU were willing to attend ICT course, whereas at GXMU about two-third (20 out of 32) and at FDU about one-third (3 out of 9) were willing to do so. A time constraint was the main reason for students’ unwillingness to attend ICT courses. One student said:

I want to attend more courses on the application of ICT, but I may not have time to attend regularly...

A participant added:

I did attend a ICT course when I was a freshman, but I forgot most of the details since lack of practice after class,butIsuppose the use of ICT can help a lot in my study.

### Understanding About the Pros and Cons of Web-Based Training

Students’ impression about the idea of a Web-based course, in general, was mostly positive. Students thought that the access to varieties of lectures from prestigious universities including Ivy League schools would enrich their learning experience and professional knowledge. The flexibility and convenience along with abundant content were mentioned as the common advantages in the FGDs.

Several common disadvantages of Web-based courses were mentioned by students across the universities: lack of interactions, poor quality and out dated content, and the high price to attend the course. One graduate student mentioned:

Online course do possesses a large amount of advantages, for instance, it is time-saving and labor-saving,out of the restriction of location and time. But, sometimes,Iwas lost in thelargenumber of online courseavailable,Icannot select out the ones that were most useful to me and my professional development.

Another student added:

Online course lacks face-to-facecommunicationswith teachers...alsoour confusion cannot be solved instantly.

**Table 1:** Demographic characteristics of focus group discussion (FGD) participants (N=74).

Demographic characteristics		GXMU^a^ (n=32)	GXTCMU^b^ (n=12)	GLMU^c^ (n=7)	GZHMU^d^ (n=7)	KMMU^e^ (n=7)	FDU^f^ (n=9)
Focus group discussion date		November to December, 2015	March 25, 2016	March 21, 2016	May 20, 2016	April 19, 2016	March 28, 2016
**Gender**							
	Male	11	9	0	4	1	1
	Female	21	3	7	3	6	8
Age, mean (SD^g^)		24.56 (1.50)	26.84 (3.74)	25.71 (0.49)	25.43 (2.94)	25.14 (1.21)	26.64 (0.67)
Public health		11	2	0	7	7	4
Clinical medicine		6	7	7	0	0	5
Basic science		6	2	0	0	0	0
Others (ie, nutrition, pharmacology)		9	1	0	0	0	0
**Grade of study**							
	First-year graduate	18	10	0	4	4	3
	Second-year graduate	8	2	7	3	2	3

^a^GXMU: Guangxi Medical University.

^b^GXTCMU: Guangxi University of Chinese Medicine.

^c^GLMU: Guilin Medical University.

^d^GZHMU: Guangzhou Medical University.

^e^KMMU: Kunming Medical University.

^f^FDU: Fudan University.

^g^SD: standard deviation.

**Table 2:** Typical statements made by participants by key themes.

Themes	Examples of typical statements made by the participants
Attitudes toward ICT^a^	A tool for learning knowledge; the development of software; the spread and promotion of technology as well as new information; supplementary means to improve the efficiency in the field of scientific research.
	A mean of sharing information and getting the newest information; the technology that offers convenience to all society members.
	A form similar to online-to-offline (O2O) pattern which was put forward by Jack Ma; information dissemination, storage, analysis, and transmission in the era of big data.
	Electronic equipment related to computer; offer help to scientific research and life with science and technology equipment to; tools for electronic information transmission.
	The category from daily email to literature retrieval and paper check in the field of graduate students’ research topics; a perfect combination between communication and information technology.
	The way to get information from the Internet (statistical software, information retrieval, mobile phone app, Google Glass, Leonardo’s Robot, and so on)
ICT application in routine research	Searching and reading electronic journal and papers (ie, PubMed); academic teleconference; translate the latest and updated foreign publications; online courses relevant to different research methods or educational programs to support professional development.
	Software in research, such as the software for monitoring and calculating the time of apoptosis; monitoring system about punching in and out in the laboratory.
	Patients’ health data and information can be uploaded and stored to the hospital by using ICT; launch and feedback of questionnaires; long-distance diagnosis and treatment as well as teaching.
	Some apps relevant to the information of patients with AIDS^b^; the application of GIS^c^ system in scientific research; fingerprint attendance, and so on.
	Mobile phone and email, GIS, WeChat subscription in the field of scientific research; relevant software about prediction of experimental results; e-learning; MED Analysis; online English course.
	Fitness app; operation robots; some large medical equipment for health care treatment.
Understanding about the pros and cons of Web-based training (advantages)	Convenient and out of the restriction of location and time.
	Contents are rich and could be learned repeatedly.
	It’s possible for students to have online courses based on their own arrangements; promote resources sharing and academic exchanges.
	Online courses are helpful to improve educational equity.
	Online course are time-saving and labor-saving.
	Online courses are good for resources sharing with other academicians and professionals.
	Online courses are convenient and cheap.
	Course time could be arranged by individuals; some online courses could be downloaded based on individual interests.
Understanding about the pros and cons of Web-based training (disadvantages)	Ask interactive communication; not good for weak willpower learners as they easily got absent-minded.
	Some online-courses are expensive.
	Some free online courses are free of time limit, students are prone to become lazy.
	Lack of academic atmosphere; not possible to raise questions after a course.
	There are too many online courses and it is difficult to pick up appropriate courses.
	It’s difficult for students to overcome their laziness and persist in learning all courses.
	Commercial advertisements are added in some online courses which direct users to nonrelevant sites.
	Students are interested in online courses at the beginning, but they lose their interests quickly due to the vagueness of courses and lack of professional answers to problems.
Attitudes toward the design of ICT training curriculum	Course contents should be authoritative and classified enough for different majors; the length of course time could be arranged individually based on students’ interests.
	It’s easy to learn lessons by exploring medical websites; it’s better to have interactive questions and answers after classes; forms of ICT training courses should be flexible.
	It’s better to have ICT training courses on the Internet; the time of an online ICT training course should not be over 20 min, whereas the time of a practice course could be extended appropriately.
	Traditional methods and theories should be combined with practices; ICT training courses would not be selected by students willingly if the conduct of ICT training courses is linked to students’ academic degrees and credits because the effect of being forced to learn is always less satisfactory.
	The course time and main content even schedule totally depend on individual’s choices.
	The courses with hands-on practices are more interesting; it’s more important to introduce the approaches and solutions to solve problems than merely delivering theories.
Potential challenges to promoting ICT courses	Whether ICT or ICT training courses are relevant to difficult majors and can cater students’ interest; to promote ICT training courses, we could deliver lectures for targeting potential students accurately.
	There will be competitions which come from the similar brand courses when promoting courses at the beginning.
	By giving gifts at the very beginning and showing the greatest benefit of the courses or the new technologies. Provide some demo courses and show feedback of user experience.
	Concerns about course’s cost; the authority of the courses’ contents.
	Due to the reliance on electronic technology, the loss of data would be irreparable if the system collapses.
	Some software are blocked due to copyright; people may consider to learn ICT only when it is needed because of the limit of time and energy; the quality of the electronic questionnaires cannot be confirmed.
Marketing strategies for ICT training curriculum	Take advantage of celebrity effect and employ the renowned professors to promote the course.
	Shows people the successful experience of previous course and ICT application in research.
	The titles of the courses should be understood easily and eye-catching to raise interest.
	By playing short videos about the course and explaining the benefits.

^a^ICT: information and communications technology.

^b^AIDS: acquired immunodeficiency syndrome.

^c^GIS: geographic information system.

### Attitude Toward the Design of ICT Training Course

When asked about the design of ICT training courses, opinions differed across the universities. Some students were in favor of more organized training courses whereas some were in favor of short seminars or lectures. When asked about desired forms of teaching methods for ICT training course, one participant said:

It would be better if traditional teaching methods were combined with the modern method (ie, an online course), we would welcome some novel class formats such as workshop, new lecture-delivery methods, group discussions.

Several students thought that the length of a course and the time required should be specified according to students’ major and areas of research.

One of the participants mentioned:

If a course is useful for my major and research,Ifeel obliged to learn the course material again and again untilIhave acquired the skills being taught.

When asked whether a course should be linked to a student’ academic degree and credits, 68 out of 74 graduate students (92%, 68/74) were strongly against it. One of the students stressed:

ICT courses are always less satisfactorywhenbeing forced to learnin a formal classroom setting.

One participant voiced concern:

Having access to a computer (desktop orlaptop)is a necessary step to using ICT, but some student who come from poorer backgrounds cannot afford buying a computer,Iguess this is the priority.

### Potential Challenges and Strategies for ICT Course Promotion

The participants in FGDs listed a series of unfavorable factors: whether the course is relevant and pertinent to each individual’s majors, how the grading in the ICT course is linked to their overall academic grading, and best practice of other universities related to ICT courses.

For example, a student said:

Tied in heavily on daily schedule, we would think twice whether we accept an ICT course or not.

Another student added:

If the ICT course is not relevant to my current research, Iwould definitely pass it, we are too busy to burden too many tasks.

One of the participants doubted:

Is there rich evidence showing that the ICT made huge contribution to medical research than any other field?

Apart from above statements, an ICT course’s financial expenditure and time costs were also emphasized by 35 out of 74 participants (47%, 35/74) in the focus groups.

### Marketing Approaches for ICT Courses

Few students (9 out of 74) suggested approaches to promote ICT courses, such as posting advertisements at university websites and employing professional experts to market the course. One graduate student suggested:

In order to promote ICT course among students, we could rely on various types of social media such as Wechat, Weibo (China’s version of Twitter),and publicizing posters around the university campus.

A participant stressed:

To the best of my knowledge, feedback from former student is the most useful way to popularize an ICTcourse,therefore engaging former students in the promotion of a new ICT course would be useful.

Several students deemed that it is necessary to set some ICT course as a required course for graduate students. They said:

Some students won’t spend time on any course unless they realized it is useful or the course is a required course.

In addition, 12 out of 74 students (16%, 12/74) suggested providing demo classes to target students and offering free lectures for the entire school as ways to promote a new ICT course.

## Discussion

### Principal Findings

This study is among the first in China that explored the nature and need for ICT-related training for research focused graduate students at medical universities in China. This study informed us of the situation and level of implementation of ICT use-related training at these medical universities. Whereas the participants’ understanding and attitudes varied from individuals to individuals, this documented important information about the nature of ICT training that would be applicable to graduate students engaged in biomedical or population-based research. Based on our findings, the usefulness of ICT for research focused graduate students lies in the ability to collect and analyze data, synthesize information from the Internet, and for use in teleconferencing. Participants identified some popular methods they had used on a daily basis in their research such as software used to identify information related to patients with chronic diseases, and geographic information systems (an information system that is used to input, store, retrieve, manipulate, analyze, and output geographically referenced data or geospatial data). Information sources included Wechat subscriptions and PubMed, which were used for identifying and tracking current and past research findings, conducting questionnaire-based surveys, and for analyzing experimental results. Early studies also reported that health-oriented social networking groups may represent a future for health care, medical practice, and medical research that is radically different from those used several decades ago [17,18].

Current advances in ICT that have been transforming our society have tremendous potential to improve health care in areas such as administrative and clinical care, consumer health, biomedical and health services research, financial transactions, professional education, and public health. The participants stressed their strong willingness to participate in highly specialized ICT courses related to their academic majors or the types of courses that would give them a better understanding of their research fields. In a study by Teresa et al [19], therapists used ICT for work management tasks and professional development. In addition, novel teaching methods (ie, workshops, group discussions) would seem to be more welcomed among graduate students if they are developed in a way that could stimulate and motivate the students as well as assist them in fostering sensible learning habits and efficient self-study methods.

Limited time and energy, value of an ICT course to individual’s research, as well as financial implications are the main challenges to promoting an ICT curriculum. With the widespread use of the Internet, the new information age has brought us a great amount of convenience in that we are able to obtain enough information about many topics with only a few simple clicks. In this study, a considerable number of participants complained that they became confused, wasted time, and became distracted by an incredibly large quantity of information available when they searched on the Web. These results confirmed a previous finding that magnanimous information and a bewildering variety of Web links have occupied much of our time and energy [20,21]. Therefore, it is quite understandable that graduate students would think twice when a new curriculum or new course pops up and becomes available. Considerations such as how long would a course of study last and what are the fee standards involved will require considerable discussion. Novel ICT curriculum with highly specific and pertinent information related to their major would be more welcomed by already time-stressed graduate students in the health sciences field. Apart from what we discussed above, the copyright of specific software and cultural factors may also affect the promotion of ICT curricula [22-24].

### Strengths and Limitations

The strengths of our study were not only the diverse range of respondents involved in the study in terms of specialty, gender, grade, work experience, and location (6 different cities in southern China), but also by the participation of research focused graduate students from different medical universities and health sciences schools that allowed us to gather varying views. One limitation was that all participants were recruited from only 6 cities in southern China, limiting our ability to generalize the findings to attendees in other areas of China. However, this study represents the first known qualitative study that focuses on the thoughts and attitudes of ICT use and ICT training among research focused graduate students studying in different areas of health sciences field in China. The findings should encourage more research in this area with the goal of promoting the development and use of ICT curriculum to promote health sciences research in LMICs.

### Conclusions

This is the first qualitative study in China, which reflects the perceptions and needs of research focused health sciences graduate students toward ICT and its expanding use in biomedical and population-based research and training. The findings highlight the graduate students’ demand for organized ICT curriculum along with the positive and negative aspects of currently available ICT tools (ie, Web-based education programs). Whereas a need exists for a nation-wide survey to better understand ICT use among graduate students engaged in health sciences research across medical universities throughout China, the current local findings provide some basis that could be used in the development of a training program or a model curriculum to be used by graduate students engaged in biomedical or population-based health research.

## Abbreviations

AIDS: acquired immunodeficiency syndrome
FDU: Fudan University
FGD: focus groups discussion
GIS: geographic information system
GLMU: Guilin Medical University
GXMU: Guangxi Medical University
GXTCMU: Guangxi University of Chinese Medicine
GZHMU: Guangzhou Medical University
ICT: information and communications technology
KMMU: Kunming Medical University
SD: standard deviation
